# Ovarian Cyst Fluid as a Resource for Diagnostic and Prognostic Biomarker Discovery in Ovarian Lesions

**DOI:** 10.3390/cancers18193182

**Published:** 2026-10-02

**Authors:** Lamia Sabry Aboelnasr, Nanki Deol, Yingran Jin Fu, Mona El-Bahrawy

**Affiliations:** 1 Department of Metabolism, Digestion and Reproduction, Faculty of Medicine, Imperial College London, Hammersmith Hospital, London W12 0HS, UK; l.aboelnasr22@imperial.ac.uk (L.S.A.); yingranjinfu@gmail.com (Y.J.F.); 2 Department of Pathology, Faculty of Medicine, Menoufia University, Shibin El Kom 32511, Egypt; 3 Department of Pathology, Faculty of Medicine, University of Alexandria, Alexandria 21131, Egypt

**Keywords:** ovarian tumour microenvironment, cyst fluid, biomarkers, proteomics, metabolomics, glycoproteomics, tumour DNA, diagnosis, ovarian cancer

## Abstract

Background/Objectives: Differentiating benign, borderline, and malignant ovarian cystic lesions before surgery remains challenging, and many women undergo operations for lesions that prove benign. Most cystic lesions contain fluid that lies in direct contact with the lesion and forms part of the tumour microenvironment, potentially concentrating tumour-derived molecules diluted in the circulation. This review presents the current evidence on the value of ovarian cyst fluid analysis for the discovery of diagnostic and prognostic biomarkers across benign, borderline, and malignant lesions. Methods: We reviewed studies analysing ovarian cyst fluid using proteomic, glycoproteomic, metabolomic, inflammatory, and tumour-DNA approaches, together with conventional biomarkers and cytology, and considered pre-analytical and methodological factors affecting their interpretation. Results: Cyst fluid harbours proteins, glycoproteins, metabolites, cytokines, and DNA with tumour-specific mutations, frequently at concentrations exceeding those in matched serum. Multiple candidates discriminate malignant from benign lesions, and borderline lesions often show intermediate signatures. No single marker outperforms established CA125-based protocols, but multi-marker and multi-omics combinations have shown improved diagnostic performance within individual studies. Conclusions: Ovarian cyst fluid is a biologically enriched, underexploited substrate and a potential adjunct to existing diagnostic pathways. Realising its clinical potential will require standardised pre-analytics, validated multi-analyte panels, and prospective studies addressing clinical utility and reproducibility.

## Simple Summary

Ovarian cysts are very common, and most are harmless. However, some are cancerous or may become cancerous, and telling these apart before surgery remains difficult. Many women therefore undergo operations for cysts that turn out to be benign. Most cysts contain fluid, which sits in direct contact with the cyst wall and may carry molecules released by the cells lining it. This review gathers the current evidence on what ovarian cyst fluid contains and whether analysing it could help doctors distinguish benign, borderline, and cancerous cysts. Studies show that cyst fluid holds proteins, sugars, small metabolites, and tumour DNA, often at higher levels than in blood. No single molecule is yet reliable enough for clinical use, but combining several types of measurement appears more promising. With standardised collection and further testing, cyst fluid analysis could support earlier, less invasive diagnosis.

## 1. Introduction

Approximately one in five women will develop an ovarian mass or cyst in their lifetime, the majority of these being benign [1,2,3]. Ovarian cysts are fluid-filled sacs that develop in the ovary for pathological or physiological reasons, most commonly during the natural menstrual cycle [1]. They encompass a broad spectrum of entities, including functional cysts, endometriomas, and benign, borderline, and malignant tumours [2,4]. A clinically important subset, however, represents borderline or malignant tumours [2,4,5].

Tumour tissue examination is the diagnostic gold standard and gives insight into tumour biology, but representative tissue samples may not capture intratumour heterogeneity [6]. Ovarian cyst fluid analysis may provide additional valuable information, as the fluid resides in immediate proximity to tumour tissue, and represents part of the tumour microenvironment (TME). As such, it may contain a concentrate of tumour-derived proteins, metabolites, glycans, cytokines, and nucleic acids that are diluted in systemic circulation and not detectable in blood at early stages of tumour development and progression [7]. Elevated inflammatory mediators in cyst fluid relative to matched serum samples further support that this compartment reflects local tumour biology rather than systemic spillover [8]. Despite their potential, cyst fluid remains underexplored [7,9].

This review presents the current status of the value of molecular and biochemical profiling of cyst fluid from benign, borderline and malignant lesions, focusing on diagnostic potential and mechanistic insight into tumour progression and metastatic potential.

## 2. Materials and Methods

### 2.1. Literature Search

This narrative review draws on a structured search of PubMed/MEDLINE, Embase, Scopus, and Web of Science Core Collection, from database inception to March 2026. Search terms relating to ovarian and adnexal lesions (“ovarian cyst”, “ovarian mass”, “adnexal mass”, “ovarian tumour”, “ovarian neoplasm”, “ovarian cancer”) were combined with terms describing cyst fluid and related specimens (“ovarian cyst fluid”, “cystic fluid”, “tumour fluid”, “cyst aspirate”, “cyst aspiration”), together with terms for the principal molecular and diagnostic domains covered here: biomarkers and tumour markers; proteomics and mass spectrometry; cytokines, inflammation and angiogenesis; metabolomics; glycomics and glycoproteomics; tumour-derived DNA, cell-free DNA, somatic mutations and genomic sequencing; conventional markers such as CA125, HE4 and CA19-9; and cyst-fluid cytology. Pre-analytical factors, including sample collection, processing, storage, centrifugation, freeze–thaw cycles and assay standardisation, were searched separately. Reference lists of relevant articles were screened manually and key publications were followed forward through citation searching. We focused on peer-reviewed primary studies characterising the molecular, biochemical, cytological, diagnostic or prognostic properties of ovarian cyst fluid across benign, borderline and malignant lesions, and appraised the strengths and limitations of individual studies, including cohort size, histotype representation and validation status, throughout the review.

### 2.2. Pre-Analytical Considerations

Pre-analytical factors are a major and frequently underappreciated source of variability in cyst-fluid studies, and inconsistent reporting of these variables limits reproducibility and cross-study comparison. Across the studies reviewed, cyst fluid was most often aspirated after surgical removal of the cyst, centrifuged to remove cells and debris, aliquoted, and stored at −80 °C, typically within 30–60 min of collection and after a single freeze–thaw cycle [7,9]. Even within this broadly similar workflow, several variables differ between studies and can materially affect molecular readouts.

Haemorrhagic and blood contamination. Many cyst fluids, particularly endometriomas and some malignant cysts, are heavily blood-stained, and abundant plasma proteins (albumin, immunoglobulins) can dominate proteomic spectra and mask lower-abundance, tumour-specific signals; several studies therefore apply abundant-protein depletion before mass spectrometry, and some exclude grossly haemorrhagic or highly viscous samples altogether [7,9,10]. Because CA125 and many cytokines are also present in blood, contamination with blood or with ascitic fluid can artefactually raise measured concentrations and confound comparison with serum [11]. The degree of blood contamination is rarely quantified, yet it directly affects proteomic and metabolomic profiles.

Analyte stability and storage. Different analyte classes have different pre-analytical sensitivities. Proteomic and metabolomic readouts are affected by centrifugation conditions, abundant-protein depletion, and repeated freeze–thaw cycles [9]; labile cytokines are sensitive to processing delay and freeze–thaw, so time from collection to freezing is an important, and often unreported, variable. Tumour-derived DNA appears comparatively robust; Wang et al. recovered a median of 222 ng of DNA per cyst and could not sequence only a small number of samples with insufficient (<3 ng) DNA, though yield varies widely and depends on cellularity [12]. Standardising centrifugation speed and timing, the number of freeze–thaw cycles, and storage temperature is therefore essential for reproducibility.

Cyst size, viscosity and sampling. Cyst size and fluid viscosity influence both the ease of sampling and the representativeness of the aspirate; mucinous and older endometriotic cysts yield highly viscous fluid that is technically difficult to handle and process, whereas thin serous fluid is more readily analysed [7]. Very small cysts may yield insufficient volume or DNA for some assays.

Standardisation recommendations. On the basis of the available evidence, future cyst-fluid studies should, at minimum, report and, where possible, standardise: the timing and mode of collection; the interval from collection to processing and freezing; centrifugation speed and temperature; the number of freeze–thaw cycles; storage temperature; and an objective measure or grading of blood contamination. Adoption of such minimum reporting standards would substantially improve the comparability and reproducibility of cyst-fluid biomarker research.

## 3. Types of Ovarian Lesions and Previous Studies Analysing Cyst Fluid in Different Lesions

It is important to emphasise that the lesions considered in this review (functional cysts, endometriomas, benign epithelial tumours, borderline tumours, and the type I and type II epithelial ovarian cancers) are biologically and clinically distinct entities rather than points on a single continuum. Borderline tumours in particular should not be regarded merely as an intermediate stage between benign and malignant disease: although some (notably serous borderline tumours harbouring BRAF or KRAS mutations) can be precursors of low-grade (type I) carcinoma, many follow an indolent course and do not progress, and their molecular features differ from those of high-grade (type II) cancers. Molecular alterations in cyst fluid are therefore best interpreted in a histotype-specific context, and comparisons across lesion categories should account for these fundamental biological differences.

### 3.1. Ovarian Cysts

Ovarian cysts typically fall into two overarching categories: functional or pathological. Functional cysts develop during the menstrual cycle, and include follicular, corpus luteum, or theca lutein cysts [1,2,5].

### 3.2. Functional Cysts

During a typical menstrual cycle, rising follicle stimulating hormone (FSH) leads to a dominant follicle housing the oocyte and cumulus cells. A luteinising hormone (LH) surge triggers ovulation, releasing the oocyte–cumulus complex; the follicular remnant becomes the corpus luteum, which raises progesterone to prepare the endometrium for implantation. If fertilisation does not occur, the corpus luteum disintegrates [13].

#### 3.2.1. Follicular Cyst

If the follicle fails to rupture, a follicular cyst can form. Failure to release the oocyte can be due to insufficient levels in FSH or LH. These cysts grow due to continued hormone stimulation and are benign [1,2]. Typically, these cysts can resolve within a few menstrual cycles without outside intervention.

#### 3.2.2. Corpus Luteal Cyst

If the corpus luteum fails to lyse during the menstrual cycle, a corpus luteal cyst can form. Like follicular cysts, these cysts are typically benign and can resolve on their own. Women who are pregnant will have corpus luteum cysts in the first trimester, but these typically resolve spontaneously [1,2].

#### 3.2.3. Theca Lutein Cyst

Theca lutein cysts arise during pregnancy and are triggered by an increase in human chorionic gonadotropin (hCG) amount or sensitivity. These cysts form luteinised follicles and are usually present in complicated pregnancies [13].

From a clinical standpoint, functional cysts are the most common ovarian cystic lesions and are central to the differential diagnosis of an adnexal mass. They are typically thin-walled, unilocular, and anechoic on ultrasound, and the majority resolve spontaneously within one to three menstrual cycles, so that expectant management is usually appropriate. Their fluid reflects this benign, physiological origin: follicular and luteal fluids are hormonally active, containing the steroid hormones (oestradiol, progesterone) and gonadotropin-regulated proteins of normal folliculogenesis, rather than the tumour-associated proteins, inflammatory cytokines, glycan alterations, and somatic mutations that characterise neoplastic cyst fluid. Because functional cysts are so common and are frequently sampled incidentally, their fluid provides a physiological benign comparator against which tumour-associated changes can be judged, and care must be taken not to misattribute normal folliculogenesis-related signals to neoplasia. Reliable pre-operative discrimination of functional from neoplastic cysts, using validated sonographic models such as the IOTA Simple Rules together with clinical and biochemical assessment, is therefore a prerequisite both for appropriate management and for meaningful interpretation of cyst-fluid molecular data [14].

### 3.3. Pathological Cysts

Pathological ovarian cysts are cysts that do not form during the menstrual cycle. Pathological cysts include endometriotic cysts or tumours, which may be epithelial tumours, sex cord stromal tumours or germ cell tumours.

#### 3.3.1. Endometriomas

Endometriomas are cysts associated with endometriosis. The fluid within these cysts has been studied in order to date these lesions, as well as understand the differences between those that are benign versus ones that are associated with neoplastic components. Endometriomas are distinctly known for their fluid colour, oftentimes taking a deep brown colour, referred to as “chocolate” fluid due to the accumulation of blood within the cyst [7,15]. This reflects repeated injury and haemorrhaging into these cysts, with accumulation of erythrocytes within the fluid. Older endometriotic cysts contain more viscous fluid with higher iron content [15], consistent with greater injury and bleeding over time. Additionally, older cysts were found to contain fluids higher in bilirubin and ferritin, both of which coincide with high iron levels [7,15]. Analysing endometrioma cyst fluid has allowed researchers to identify differences between young and old lesions, which in turn can allow clinicians to navigate treatment depending on the cyst age.

Treatment of endometriomas can involve hormone therapy, surgical removal, or both, and patients undergoing operations to remove the cysts often have a recurrence in their symptoms, which can be mediated using oral contraceptives [16]. However, in patients who are either unable to take oral contraceptives, or choose not to, cyst recurrence rates can reach 27% in the two years following the operation [17]. Surgical techniques for endometrioma removal follow two paths: removal of cyst fluid prior to lesion removal, or simultaneous suctioning of cyst fluid and lesion removal. Both techniques pose the risk of cyst fluid leakage into the surroundings, which may lead to recurrence. The presence of live endometrial cells within the endometrioma cyst fluid has illustrated a potential mechanism by which the fluid promotes recurrence [16,17].

Aside from dating endometriomas, researchers have analysed the cyst fluid to differentiate benign masses from endometriosis-associated ovarian cancer (EAOC). EAOCs possess much lower iron concentrations (free iron, heme iron, and total iron) within their cyst fluid in comparison to benign endometriomas, with a diagnostic cut-off of 64.8 mg/L [15]. MR relaxometry was then investigated for measuring iron in vivo. In vivo concentrations within EAOCs and endometriomas correlated positively with ex vivo measurements, and the technique distinguished the iron difference between the two, suggesting potential for non-invasive malignant detection [4,8,15]. Further studies demonstrated that MR relaxometry distinguishes malignancies from endometriosis with a sensitivity and specificity of 80.6% and 75% (*p* < 0.001) respectively, when classifying atypical and borderline tumours as EAOCs [15]. This estimate derives from a single study and required atypical and borderline tumours to be grouped with malignancy, so it awaits independent confirmation.

Along with iron concentrations, benign endometriomas possess higher levels of methaemoglobin (metHb) in their fluid compared to EAOCs [8,15,16]. Compared to haemoglobin (Hb) or oxyhaemoglobin (oxyHb), which contain iron in its Fe^2+^ state, metHb contains Fe^3+^, the oxidised version of iron. Hb iron oxidises to metHb iron following erythrocyte lysis, which occurs repeatedly within endometrioma cyst fluid. This auto-oxidation also produces free radicals and reactive oxygen species (ROS), both associated with cell and DNA damage. High metHb within endometrioma cyst fluid may therefore correlate with high ROS and free radical concentrations, illuminating a possible mechanism of malignancy [18]. A later study demonstrated that endometrioma cyst fluid contains high levels of ROS that, when incubated with endometriotic epithelial cells ex vivo, induce oxidative stress [18,19].

The ratio of metHb/oxyHb was shown to be significantly higher in endometriomas compared to EAOCs. Moreover, EAOCs also possess significantly lower bilirubin levels than endometriotic cysts. When looking at bilirubin alongside iron concentrations, a positive correlation was found between the amount of free and total iron and the amount of bilirubin in EAOCs, but not in benign endometriotic cysts [8,18]. More recently, the mechanisms behind the effect of endometrioma cyst fluid on the surrounding tissue have been studied. Ye et al. discovered that endometriotic cyst fluid may activate the cGAS/STING pathway within ovarian tissue [20]. This pathway activation was associated with malfunctions in mitochondria and inflammation [19,20]. These findings emphasise how analysing the profile of endometrioma cyst fluid has greatly illuminated the characteristics of these cysts, as well as potential mechanisms behind their malignant transformation.

#### 3.3.2. Polycystic Ovary Syndrome

Polycystic ovary syndrome (PCOS) is an endocrine disorder that can result in the presence of fluid-filled follicles [21]. These sacs form due to stunted follicle growth, leading researchers to investigate the composition of follicular fluid within PCOS patients to identify any mechanisms that may contribute to the sac formation. Proteomic analysis of PCOS follicular fluid uncovered a wide range of expressed glycoproteins that were not present in normal follicular fluid, including an upregulation in SERPINA1 and downregulation in ITIH4 [21]. These proteins contribute to angiogenesis and the extracellular matrix, indicating potential mechanisms by which they alter follicle health [21]. MMP-2 and MMP-9 and their inhibitors TIMP-1 and TIMP-2 were also found to be expressed at significantly higher levels in PCOS patients undergoing IVF compared to women with normal ovaries. These proteins contribute to follicular matrix formation, and their upregulation signals a potential pathway by which PCOS follicles remodel [21].

Follicular fluid contains granulosa cells, which contribute to abnormal follicle behaviour when they abnormally proliferate. miR-145 was discovered to be downregulated in granulosa cells from PCOS follicular fluid, which correlates with high proliferation and inhibited apoptosis [21,22]. Moreover, miR-145 was discovered to inhibit insulin receptor substrate 1 (IRS1), which inhibits granulosa cell proliferation. Therefore, low miR-145 levels lead to IRS1 activation, promoting granulosa cell proliferation in PCOS patients [22]. These findings have illuminated potential targets in PCOS follicular fluid that could aid in the management of follicular cysts.

Beyond these follicular-fluid findings, it is important to place them in clinical context. PCOS is a common endocrine and metabolic disorder, diagnosed by the Rotterdam criteria (two of three features: oligo-/anovulation, clinical or biochemical hyperandrogenism, and polycystic ovarian morphology, with exclusion of related disorders), and is associated with insulin resistance and an increased long-term risk of type 2 diabetes and cardiovascular disease [23]. Its “cysts” are in fact arrested antral follicles rather than true cysts, and management is directed at the clinical phenotype (lifestyle measures, ovulation induction, insulin-sensitising agents, and cycle regulation) rather than at the follicles themselves. The follicular-fluid alterations described above are therefore of mechanistic and potential prognostic interest, for example in understanding impaired folliculogenesis and IVF outcomes [21,22], rather than diagnostic targets in the sense applied to neoplastic cyst fluid. Distinguishing the multiple small follicles of PCOS from pathological cystic lesions is nonetheless part of the clinician’s differential, underscoring the value of accurate characterisation of ovarian cystic fluid across the full spectrum of lesions.

### 3.4. Ovarian Neoplasms

The World Health Organization (WHO) classification 2020 of ovarian tumours includes principally epithelial, mesenchymal, sex cord stromal, and germ cell tumours listed in Table 1 [24]. Within each of these categories there are tumour subtypes, including benign and malignant tumours as well as borderline tumours or tumours of potential malignant behaviour.

Table 1, ovarian tumour classification according to the World Health Organization (WHO) 2020 classification, categorises epithelial, mesenchymal, sex cord stromal and germ cell tumours.

The current diagnostic measures employed for ovarian neoplasms involve visualisation through transvaginal ultrasound, MRI, or CT [25]. Accompanying these techniques is a blood test measuring known tumour markers, particularly Cancer Antigen 125 (CA125) and Human Epididymis protein 4 (HE4) [25,26]. CA125 has been widely studied for its role in malignant ovarian tumours and has been the primary biomarker used to detect malignancy. The utility of CA125 is limited, however, due to both a low sensitivity for early detection and increased levels in conditions other than malignant ovarian cancer, as underscored by large-scale screening trials [25,27]. Similarly, HE4 has been shown to increase the specificity of a malignant diagnosis when measured in combination with CA125 [25,26], while other studies indicate that the addition of HE4 does not significantly enhance the ability to make a differential diagnosis [26].

### 3.5. Clinical Context and Conventional Biomarkers

Fine-needle aspiration cytology of ovarian cyst fluid has been performed for decades, primarily intraoperatively and occasionally for diagnostic clarification. Large contemporary series demonstrate that cyst-fluid cytology is highly specific but only moderately sensitive for detecting malignancy. In an 18-year institutional study including 459 cyst-fluid FNAs, specificity reached 100%, while sensitivity was approximately 54%, with borderline tumours frequently missed due to hypocellularity and sampling limitations [28]. These data establish cytology as an effective rule-in test but highlight its limitations as a standalone screening or exclusion tool, particularly for early or borderline disease.

Conventional serum biomarkers such as CA125 are also enriched in ovarian cyst fluid. Kolwijck and colleagues demonstrated that CA125 concentrations in cyst fluid are orders of magnitude higher than in matched serum samples and correlate with histology and tumour grade [11]. However, despite these striking differences, cyst-fluid CA125 did not retain independent prognostic value in multivariable analyses once tumour stage was considered [11]. This observation underscores a recurring theme in cyst-fluid research: quantitative enrichment alone does not guarantee clinical utility, and biological context is essential.

Of other serum biomarkers, cancer antigen 19-9 (CA19-9) has been studied in combination with CA125 and HE4 to enhance prognostic and diagnostic ability [25,26]. Studies have shown that CA19-9 concentrations may exceed those of CA125 in patients with borderline mucinous carcinomas and can assist in differentiating mature cystic teratomas from other benign lesions when interpreted alongside inflammatory markers such as the neutrophil–lymphocyte ratio [29]. These findings make CA19-9 a valuable target to study within ovarian tumour cyst fluid. Emerging cyst-fluid assays incorporating nanoparticle-assisted detection of CA19-9 glycovariants have demonstrated improved detection and differentiation of borderline and malignant tumours from benign, with two MUC1 antibodies showing improved CA19-9 capture, highlighting the potential value of tumour-specific glycoforms rather than absolute antigen concentration alone [30].

Other acute-phase proteins have also been investigated within cyst fluid. Haptoglobin, an inflammation-associated glycoprotein, has been studied within ovarian cancers due to its correlation with inflammation and various malignancies [31,32]. Previous research has demonstrated that haptoglobin levels are elevated in the sera of patients with malignant epithelial tumours compared to benign and were significantly associated with the FIGO grading of malignant tumours. Additionally, higher levels of haptoglobin correlated with poorer patient outcomes for those diagnosed with epithelial ovarian cancer (EOC) [31,32]. These findings led researchers to investigate haptoglobin behaviour within the cyst fluid of benign and malignant epithelial tumours. Using proteomics in combination with IHC and ELISA, haptoglobin levels were found to be significantly higher in the cyst fluid of patients with early and late-stage malignant cysts than in benign cysts. When compared to CA125, haptoglobin specificity and sensitivity were deemed a viable biomarker of malignant epithelial tumour detection [31].

Building on these findings, Perumal et al. hypothesised that haptoglobin detection within malignant epithelial tumour cyst fluid could be more clinically practical for intraoperative use if a more suitable method of detection was utilised [31]. The standard method of detection lacked sensitivity for low haptoglobin levels and was overly sensitive to cyst fluid colour. Surface-enhanced Raman spectroscopy (SERS)-based assays were tested as an alternative, and measurements of haptoglobin were compared against CA125 serum levels. Results show SERS differentiated malignant and benign epithelial tumours with a sensitivity and specificity of 94% and 91%, respectively. This high sensitivity was consistent for stages 1–3 of malignant tumours but dropped to 86% for stage 4. SERS-based assays also showed early diagnostic potential through detection of haptoglobin levels in early-stage high-grade serous carcinoma cyst fluids that differentiated them from benign lesions. Compared to CA125, SERS-based detection showed increased sensitivity for both benign and malignant tumours, indicating potential diagnostic and prognostic significance [31]. Further research on the effectiveness of SERS-based detection for cyst fluid haptoglobin levels utilised a suspended-core photonic crystal fibre SERS sensor, showing promise for increasing the reliability of the technique [33]. These figures should be interpreted with care. They derive from a retrospective, single-institution, case–control comparison of established benign against malignant cysts, and the stage-4 subgroup comprised only seven samples. Prospective evaluation in prevalence-appropriate populations is therefore required before this performance can be considered clinically established.

Another study utilised protein analysis techniques aimed at investigating the expression of specific markers, including N-acetylaspartate, inhibins, gonadotropins, and apolipoproteins. N-acetylaspartate was found to have higher expression within the cyst fluid of serous carcinoma compared to mucinous, endometrioid, or clear cell carcinomas, and presented in much higher concentrations within the cyst fluid than in corresponding blood tests [34]. Inhibin A, a protein that suppresses the release of follicle-stimulating hormone, was found in significantly lower concentrations within malignant EOC cyst fluid compared to borderline tumours. Furthermore, Inhibin A levels were significantly higher in patients with a better prognosis and life expectancy [34]. These findings correspond with FSH levels within epithelial malignant tumours. Malignant tumours had significantly higher levels of FSH and LH within their cyst fluid compared to benign lesions, with LH and FSH levels also correlating with the FIGO stage of the tumour [34]. These findings illuminate potential hormonal mechanisms behind malignancy that may be well reflected in tumour cyst fluid. Apolipoproteins were discovered to have higher concentrations in EOC cyst fluid than in benign tumours, and further validation through ELISA demonstrated that apoA2 and apoE may have potential for differentiating malignant from benign or borderline tumours, perhaps highlighting differences in lipid metabolism that aid in malignant transformation [35].

The molecular composition of cyst fluid is histotype-dependent, yet most studies to date have examined predominantly serous epithelial tumours or have not stratified their cohorts by histological subtype. The applicability of many findings to mucinous, endometrioid and clear-cell tumours therefore remains uncertain, and the histological context of individual studies is noted throughout where it is reported.

Because CA125, alone or combined with HE4 in clinical algorithms such as the Risk of Malignancy Index (RMI) and the Risk of Ovarian Malignancy Algorithm (ROMA), remains the clinical reference standard against which any new marker must be judged [25], it is worth stating explicitly what would constitute genuine added value. A candidate marker is most useful if it provides information independent of CA125 rather than merely correlating with it; if it raises diagnostic accuracy when incorporated into a multi-marker model; if it improves sensitivity, particularly in early-stage disease where CA125 most often fails, without an unacceptable loss of specificity; if it helps with clinically difficult distinctions, especially borderline from malignant disease; and if it adds to, rather than duplicates, the information already available from imaging.

Judged against these criteria, several cyst-fluid markers show early promise while still falling short of established utility, and the relevant study populations, methods, and validation status for each are summarised in Table 2. The clearest examples of independent, additive value come from studies that tested markers alongside CA125 directly: apolipoprotein C-III with protein C inhibitor emerged as independent predictors of malignancy and, combined with serum CA125, raised the area under the curve to 0.94 and lifted specificity from 68% to 88% relative to CA125 alone [7], while SERS-measured haptoglobin exceeded the sensitivity and specificity of CA125 in the same cohort [31]. Tumour-derived DNA is attractive precisely because its specificity is mechanistically independent of CA125, since driver mutations mark neoplasia directly rather than reflecting the non-specific elevations that limit CA125, and cell-free DNA approaches have repeatedly outperformed CA125 in early-stage disease [36]. Several proteomic and metabolomic panels likewise report improved discrimination over CA125 within their cohorts, though these gains are drawn largely from case–control comparisons of established malignant against benign disease and require confirmation in prospective, prevalence-appropriate populations before their independent value can be regarded as proven. Performance in the borderline-versus-benign and borderline-versus-malignant distinctions, arguably the most clinically valuable and least well served by CA125, remains the least studied and is a priority for future work.

The molecular composition of cyst fluid also varies with tumour histotype, and this heterogeneity deserves emphasis. Mucinous tumours produce characteristically viscous, mucin-rich fluid in which CA19-9 is often markedly elevated, whereas serous lesions yield thinner fluid; N-acetylaspartate, for example, is higher in the cyst fluid of serous carcinomas than in mucinous, endometrioid, or clear-cell tumours [34], and endometriomas are distinguished by their iron-, methaemoglobin-, and reactive-oxygen-species-rich contents. These differences are not merely biochemical curiosities. Fluid viscosity and debris can render some assays unreliable in mucinous and endometriotic cysts; cyst-fluid lactate dehydrogenase, a simple and inexpensive marker that discriminates malignant from benign masses reasonably well overall (90% sensitivity, 71% specificity at a threshold of 451 U/L, though with a positive predictive value of only 64%), could be measured in just a third of endometriomas in one series owing to interfering debris [46]. A practical consequence follows: a marker validated in one histotype cannot be assumed to perform equally in another. It must be acknowledged, however, that the evidence base for such histotype-specific effects remains limited, since most cyst-fluid studies either pool histotypes or examine predominantly serous disease, and few are designed to compare biomarker performance directly across histological subtypes. Systematic, histotype-stratified studies are therefore an important priority, and reported performance should meanwhile be interpreted in light of the histological composition of the cohort from which it was derived.

### 3.6. Metabolomics

We investigated the metabolomic profile of ovarian cyst fluid across benign, borderline, and malignant epithelial tumours using proton NMR spectroscopy [41]. Metabolites showing higher levels in malignant cyst fluid included lactate, N-acetylaspartate, N-acetylaspartate glutamate, and hypoxanthine [41]. Increased lactate expression in malignant samples may indicate an increase in glycolysis, which aligns with previous findings. Additionally, unassigned peaks were consistently identified, including one at 2.03 ppm [41]. This peak was particularly prevalent in borderline spectra and may belong to N-acetyl functional groups. Citrate was present in significantly higher amounts among benign samples compared to borderline and malignant samples. Lower levels of citrate amongst malignant samples may be related to the function of citrate in cellular respiration. Because citrate is utilised in the TCA cycle and fatty acid synthesis, both of which contribute to cell growth and energy, malignant cells may be depleting citrate at a more rapid rate. Moreover, lysine levels were significantly higher in malignant samples compared to borderline or benign, which may be due to an increase in proteolysis or a decrease in oxidation.

These findings should, however, be interpreted with appropriate caution: the study was based on a small, single-centre cohort without external validation, and several NMR peaks remained unassigned. Its results are best regarded as preliminary and require confirmation in larger, independent cohorts. They are nonetheless consistent with the broader ovarian-cancer metabolomics literature (including studies in serum, plasma, ascites, and uterine fluid), which similarly implicates dysregulated glycolytic, lipid, and amino-acid metabolism and indicates that multi-metabolite panels outperform single metabolites [47,48].

### 3.7. Proteomics

Proteomics, mainly using mass spectrometry (MS), has been employed to simultaneously investigate the expression of various proteins within tumour cyst fluid. One proteomic analysis of 192 tumour cyst fluid samples (129 benign, 63 malignant) found apolipoprotein-C-III (ApoC-III) and protein C inhibitor (PCI) differentially expressed between malignant and benign samples in a mixed epithelial cohort. Neither protein alone exceeded the diagnostic sensitivity or specificity of CA125. However, when measured in combination with CA125, there was an increase in specificity and sensitivity [7]. This performance, however, was obtained by combining the markers with serum CA125 in a single-centre, case–control cohort; neither marker exceeded CA125 alone, several discriminating mass-spectrometry peaks corresponded to blood-abundant proteins such as albumin, and on independent ELISA testing only apolipoprotein C-III was confirmed, while protein C inhibitor was not. MS also detected differential expression of calgranulins within the cyst fluid of malignant/borderline tumours and benign tumours, with calgranulin A and B exhibiting higher concentrations in malignant and borderline tumours and showing some differences related to tumour stage [37].

Another study conducted proteomic analysis using iTRAQ mass spectrometry to study cyst fluid from benign serous tumours and malignant serous tumours of stage I and II. Thirty-two proteins were found to be differentially expressed between benign and malignant samples, with validation of the results confirmed through immunoblotting. Serum amyloid-4 (SAA4) was significantly higher in malignant tumour samples compared to benign. Plasma levels of SAA4 did not show significant differences between patients with benign and malignant tumours, showing that protein expression in cyst fluid can differ from that in plasma, and may be more informative [9].

Further proteomic analyses looked at the pathways associated with 75 proteins that were differentially expressed between malignant and benign tumour cyst fluid samples. Of these proteins, 49 had higher expression in malignant samples, and were associated with cell growth, cell cycle, cell communication, and cell migration. The 26 proteins that demonstrated lower expression in malignant samples were involved in metabolism and immune responses, as well as cell communication and cell migration. APOE was found to have increased expression in malignant cyst fluids [38].

More recently, the combined use of MS with liquid chromatography (TMT-LC-MS/MS) alongside machine learning models was used to identify differential expression between benign and malignant type 1 and type 2 ovarian tumours. Machine learning models were employed to analyse the proteomic landscape and identify which proteins should be experimentally validated. Verification and validation of potential biomarkers found TALDO1, ALDOA, CLU, TKT1, CP, MSLN, LAMC1, and MUC16 to be differentially expressed between benign and all malignant samples. When comparing benign samples to solely type 1 samples, only CLU is validated. The number of proteins with validated differential expression was similar when comparing benign samples to all malignant samples and to samples of type 2 tumours only. Of these proteins, ALDOA demonstrated the highest correlation between cyst fluid and serum levels and showed significant differential expression between benign serous adenoma and stage I type 1 and type 2 ovarian cancers when measured in serum [39].

While these studies were more exploratory in nature, analysing the expression of a wide number of proteins and metabolites within tumour cyst fluid, other researchers have taken more targeted approaches. These studies focused on more specific aspects of the tumour microenvironment, such as the inflammatory response and angiogenesis, for example IL-6 and IL-8 pro-inflammatory cytokines in malignant cyst fluid [8,19]. Similarly, VEGF and its soluble receptor sVEGFR-1 were observed to increase significantly in malignant and endometriotic cyst fluids compared to benign ones [43,44].

Targeted electrophoretic analysis of serum and cystic fluid has likewise identified molecular-weight-specific differences between malignant and benign ovarian cysts, including a 35 kDa protein uniquely expressed in malignant cyst fluid and serum, and a 100 kDa protein absent in malignancy, both proposed as candidate differential-diagnosis markers [40].

## 4. Ovarian Cyst Fluid as a Molecular Mirror for Tumour Progression and Dynamics

### 4.1. Inflammatory and Immune Landscape of Ovarian Cyst Fluid

Inflammation is a central driver of ovarian tumour initiation and progression, and ovarian cyst fluid consistently exhibits amplified inflammatory signalling compared with serum [49]. The cytokines IL-6 and IL-8 were found to be expressed at significantly higher levels in malignant than in benign tumour cyst fluid [8,19]. Additionally, the cyst fluid of patients with malignant ovarian masses has contained much higher IL-8 levels compared to corresponding serum levels, illustrating that IL-8 is locally enriched rather than reflecting systemic levels [19,26].

In a study investigating the expression of 15 cancer-related cytokines and growth factors within a mixed cohort of epithelial ovarian tumours, both malignant and benign tumours expressed significantly higher levels of the cytokines IL-8, MCP-1 and GRO-α in the cyst fluid compared to serum [50]. Moreover, the expression of IL-8 and GRO-α in the cyst fluid and serum of patients with malignant tumours was significantly higher than those with benign tumours. Further validation discovered differences in expression when comparing benign tumours to borderline and stage I epithelial tumours. Both borderline and malignant stage I tumour cyst fluid showed differential expression of all three cytokines when compared to benign tumours. However, while IL-8 and GRO-α levels were significantly higher in borderline cyst fluid compared to benign, this increased expression did not present in serum until stage I and III tumours for IL-8 and GRO-α, respectively [50].

Angiogenesis is another critical mechanism in tumour development and progression. Pro-angiogenic factors promote the creation of new blood vessels, therefore allowing tumours to grow and spread [43]. Vascular endothelial growth factor (VEGF), a key protein involved in angiogenesis, has been found to be expressed significantly higher within malignant ovarian tumours and endometrioma cyst fluid than in follicular cysts [43]. Further work found higher VEGF in endometrioma and carcinoma cyst fluids than in cystadenomas, indicating VEGF behaviour in endometriosis resembles malignant tumours more than benign cysts. Moreover, serum levels of VEGF did not differ significantly between the three groups. This finding is consistent with VEGF acting locally, and suggests cyst fluid may better reflect its behaviour than serum. sVEGFR-1, a receptor that regulates the angiogenic behaviour of VEGF, was studied alongside VEGF concentrations. Expression of sVEGFR-1 was significantly higher in the cyst fluid of endometriomas and carcinomas, while the ratio of VEGF to sVEGFR-1 receptors was highest in malignant tumours. This ratio indicates that malignant tumours possess higher levels of VEGF compared to its regulatory receptor. Notably, tissue measurements of malignant tumours showed low levels of VEGF and sVEGFR-1 [43,44].

Van Tilborg et al. examined fluid from benign and malignant ovarian tumours, as well as functional cysts, to analyse the differences in angiostatin and plasminogen expression [45]. Angiostatin is an anti-angiogenic protein cleaved from plasminogen, a pro-angiogenic protein. The results of this study showed that a variety of angiostatin types were expressed in all cyst fluid samples. Cyst fluids low in angiostatin tended to express low plasminogen, yet many samples expressed plasminogen and plasminogen activators with little to no angiostatin, and vice versa. Expression of the plasminogen activators uPA and tPA were found to have some relationship with the presence of angiostatin, but researchers concluded that other factors may be at play. When looking at differences between cyst fluid types, angiostatin expression was highest in malignant and borderline tumours, as well as endometriosis cysts. However, only malignant tumours consistently displayed high plasminogen levels. Functional cysts typically had moderate plasminogen levels. No strong correlations between tumour type and angiogenic protein levels emerged, though uPA and tPA may contribute to plasminogen-to-angiostatin conversion [45].

### 4.2. Proteomic Remodelling in Ovarian Cyst and Tumour Fluids

High-resolution proteomic profiling has revealed the remarkable complexity of ovarian cyst fluid. Using iTRAQ-based quantitative MS, Kristjansdottir et al. identified more than 800 proteins in cyst fluid, with 87 differentially expressed between benign and malignant serous tumours [9]. Although only two candidates were validated in follow-up analyses, this work demonstrated the breadth of tumour-associated proteomic remodelling within the cystic compartment.

Complementary work by Poersch et al. extended this approach to tumour fluid using high-throughput discovery followed by targeted multiple-reaction monitoring validation [38]. This study identified a proteomic signature distinguishing benign from malignant serous tumours, including proteins involved in extracellular matrix remodelling, complement activation, and cell adhesion such as ADAM17, CD44, SERPINF2, and SERPING1. The successful validation of multiple candidates underscores the feasibility of translating cyst-fluid proteomics into targeted diagnostic panels.

Mechanistically, the enrichment of proteases, adhesion molecules, and acute-phase proteins in malignant tumour cyst fluid aligns with known processes underlying invasion, immune modulation, and peritoneal dissemination [38]. These proteomic shifts suggest that cyst fluid not only reflects tumour presence but also mirrors functional pathways relevant to metastatic competence. More broadly, mass spectrometry-based proteomic profiling is increasingly being positioned as a route to clinically actionable biomarkers in epithelial ovarian cancer [51].

### 4.3. Glycoproteomics and Glycan-Aware Biomarker Strategies

Altered glycosylation is a hallmark of cancer and plays a key role in immune evasion, receptor signalling, and metastasis [10]. Glycoproteomic analyses of ovarian cancer fluids revealed extensive remodelling of sialylated glycoproteins. Kuzmanov et al. identified hundreds of glycosylation sites across cyst fluid, ascites, and cancer cell line secretomes, with subsets unique to cancer-associated fluids [10]. These findings implicate aberrant glycosylation as both a biomarker source and a mechanistic driver of tumour progression.

Building on this concept, Ruma et al. developed glycan-aware assay designs that move beyond antigen quantification [30]. By capturing CA19-9-bearing proteins and probing their carrier context using MUC1, MUC16, STn antibodies, and lectins, the authors achieved superior discrimination of epithelial malignant and borderline tumours from benign tumours in cyst fluid and serum compared with conventional CA19-9 or CA125 [30]. These results demonstrate that the diagnostic value of tumour markers is strongly influenced by glycan structure and carrier identity, supporting a paradigm shift toward context-aware biomarker assessment.

### 4.4. Metabolic Reprogramming Captured in Cyst Fluid

Metabolic alterations are integral to cancer progression, and ovarian cyst fluid provides direct access to tumour-associated metabolic states. Using proton NMR spectroscopy, Kyriakides et al. demonstrated distinct metabolic profiles across benign, borderline, and malignant cyst fluids, with progressive lactate and choline increases, reduced citrate in malignancy, and detection of hypoxanthine exclusively in a subset of malignant tumours [41]. These findings are consistent with enhanced glycolysis, membrane turnover, and nucleotide metabolism, features associated with aggressive tumour behaviour.

Systematic reviews of ovarian cancer metabolomics reinforce these observations. Ahmed-Salim et al. and Turkoglu et al. independently concluded that lipid and amino-acid pathways are consistently dysregulated and that multi-metabolite panels outperform single metabolites in diagnostic accuracy [47,48]. Importantly, these reviews highlight methodological challenges such as small cohorts and lack of external validation, emphasising the need for robust, integrated study designs.

Related evidence from other proximal fluids lends indirect support. In uterine fluid, Wang et al. identified a validated metabolite panel capable of detecting early-stage ovarian cancer, and in peritoneal fluid, Wojtyła et al. found that combining metabolomic and proteomic features improved diagnostic performance [52,53]. These findings reinforce the wider principle that fluids close to the tumour carry informative molecular signatures, though, coming from fluids other than cyst fluid, they bear on it only by analogy rather than as direct evidence.

### 4.5. Tumour-Derived DNA in Ovarian Cyst Fluid

Perhaps the most transformative advance in cyst-fluid research is the demonstration that tumour-specific mutations can be detected directly in cyst fluid. Wang et al. showed that mutations characteristic of ovarian cancer were present in cyst fluid from 83% of borderline tumours (19 of 23), 77% of type I cancers (10 of 13), and 100% of type II cancers (18 of 18), while no mutations were found in the cyst fluid of 18 patients with benign or non-neoplastic cysts [12].

The mutations were strongly histotype-associated, mirroring the established molecular pathology of ovarian tumours. Type II (high-grade serous) cancers were characterised almost universally by TP53 mutations, whereas borderline tumours and type I cancers harboured mutations in the RAS/RAF pathway and related genes, most commonly BRAF and KRAS, alongside NRAS, PIK3R1, PPP2R1A and PTEN. The majority of BRAF V600E mutations occurred in borderline tumours while KRAS mutations predominated at codons 12 and 61, a distribution consistent with BRAF mutation marking indolent behaviour and KRAS mutation associating with progression toward low-grade (type I) carcinoma [12]. The cyst-fluid genotype therefore reflected not only the presence of a neoplasm but also its likely histological type and aggressiveness.

Detection of these low-frequency mutations relied on a highly sensitive, error-corrected sequencing method (Safe-SeqS) applied through a multiplex panel spanning 17 recurrently mutated genes, capable of identifying mutations present in as few as ~0.1% of template molecules; categorised by the clinical need for surgery, the assay reached 87% sensitivity and 100% specificity [12]. Several features argue against a meaningful false-positive rate: no mutations were found in any benign or non-neoplastic cyst fluid, every cyst-fluid mutation was independently confirmed by a second PCR and sequencing reaction, and the corresponding tumour tissue, analysed with the same method but as material independent of the cyst-fluid assay, showed concordant mutations, with peripheral-blood lymphocyte DNA used to exclude germline and technical artefacts [12]. Independent support comes from Yamada et al., who detected tumour-associated TP53 mutations in the cyst fluid of borderline and malignant ovarian tumours but not in benign cysts, with the mutations absent from matched peripheral blood, confirming that the cyst-fluid signal is tumour-derived. Notably, their conventional PCR-sequencing approach yielded far lower detection rates (10 to 12.5%) than the error-corrected method above, underscoring the importance of assay sensitivity [54]. These findings should nonetheless be interpreted in the context of retrospective designs and modest cohorts without separate external validation.

Cyst-fluid DNA can be regarded as a proximal analogue of circulating tumour DNA (ctDNA): both interrogate tumour-derived somatic mutations non-invasively. Plasma ctDNA assays in ovarian cancer show broadly variable and often limited sensitivity, particularly in early-stage disease where tumour and mutational burden in the circulation are low [36]; cyst fluid, by contrast, lies in immediate contact with the lesion and typically carries a far higher mutant-allele fraction (median ~12.6% across surgery-requiring lesions) [12], potentially enabling detection of early lesions that shed little DNA into the blood. Because the specific mutation can indicate tumour type and pathway activation, cyst-fluid genotyping also carries potential management implications: a TP53 mutation would raise concern for an aggressive type II cancer and might favour more radical surgery, whereas a BRAF mutation would suggest an indolent borderline or low-grade lesion amenable to conservative management. Identification of actionable alterations (for example in the RAS/RAF/MEK or PI3K/PTEN pathways) could in principle inform targeted-therapy selection [12]. These applications remain to be validated prospectively but illustrate why cyst-fluid genotyping is among the most clinically compelling directions in the field.

### 4.6. Integrative Perspective and Translational Implications

**Figure 1:**
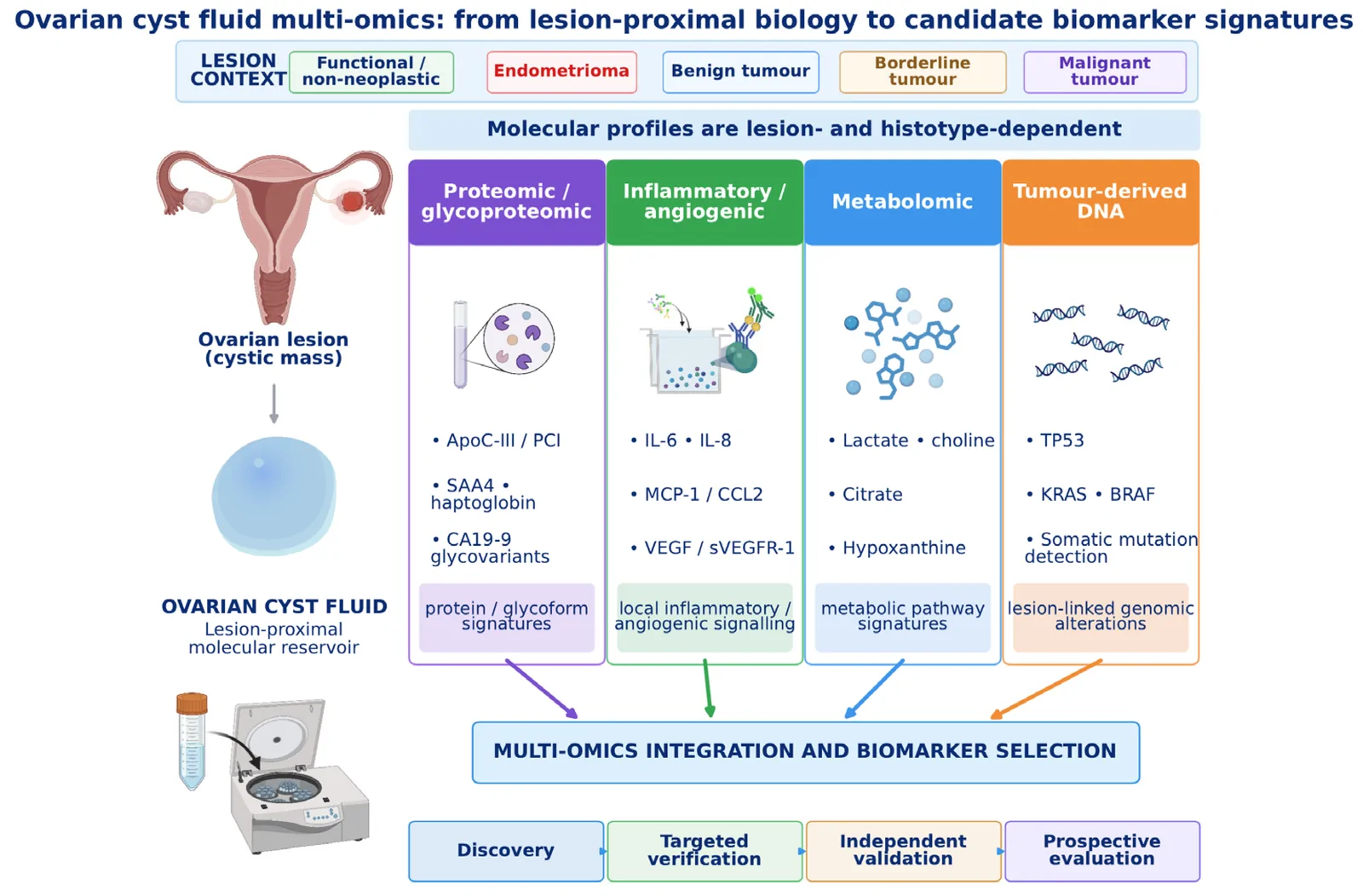
Ovarian cyst fluid multi-omics framework for biomarker discovery and validation. Ovarian cyst fluid represents a lesion-proximal molecular reservoir whose composition varies according to lesion type and histotype. Reliable analysis depends on standardised pre-analytical collection and processing. Representative candidate signals span proteomic/glycoproteomic (ApoC-III, PCI, SAA4, haptoglobin and CA19-9 glycovariants), inflammatory/angiogenic (IL-6, IL-8, MCP-1/CCL2 and VEGF/sVEGFR-1), metabolomic (lactate, choline, citrate and hypoxanthine), and tumour-derived DNA (TP53, KRAS and BRAF) domains. Integration of these complementary molecular layers provides a framework for candidate biomarker selection followed by targeted verification, independent validation and prospective evaluation; the markers shown are candidate signals requiring validation rather than established diagnostic tests. Lesion categories are presented as distinct biological contexts and should not be interpreted as a linear progression. Abbreviations: ApoC-III, apolipoprotein C-III; PCI, protein C inhibitor; SAA4, serum amyloid A4; CA19-9, cancer antigen 19-9; IL, interleukin; MCP-1, monocyte chemoattractant protein-1; CCL2, C-C motif chemokine ligand 2; VEGF, vascular endothelial growth factor; sVEGFR-1, soluble vascular endothelial growth factor receptor-1; DNA, deoxyribonucleic acid. Figure generated in Python version 3.13.5 using graphical icons created with BioRender.com.

Across inflammatory, proteomic, glycoproteomic, metabolic, and genomic domains, ovarian cyst fluid consistently captures early and amplified molecular changes associated with tumour progression. Borderline lesions frequently exhibit intermediate or early malignant signatures reflecting their malignant potential. The integration of these complementary molecular domains into a staged biomarker-development framework, from discovery through prospective evaluation, is summarised in Figure 1, and its implications for a multi-omics strategy are considered in the Discussion.

From a translational standpoint, systematic molecular analysis of cyst fluid could be incorporated without additional patient burden. Future diagnostic strategies should prioritise validated multi-parameter panels, histotype-aware interpretation, and prospective evaluation of clinical utility.

## 5. Discussion

This review presents the current evidence describing the molecular and biochemical composition of ovarian cyst fluid across benign, borderline, and malignant lesions [42,45]. Collectively, the literature demonstrates that cyst fluid represents a biologically enriched compartment reflecting the local tumour microenvironment. Multiple classes of molecular signals, such as CA19-9, occur within cyst fluid at higher concentrations than in corresponding serum, highlighting its potential to capture early tumour-associated changes and provide mechanistic insight into progression [11,55].

Although cyst-fluid analysis has not yet yielded a biomarker or signature that outperforms the current CA125 protocol, studies comparing biomarker combinations to CA125 achieved more promising sensitivities and specificities than those testing single markers [25,26], suggesting multi-marker approaches give more robust results. Though no concrete results have been clinically approved for diagnostic or prognostic use, the increase in cyst-fluid research has illuminated strong potential [8,19]. Because cyst fluid is in such close proximity to cells in a cystic ovarian mass, proteins that may act in the initial stages of tumour growth could in principle be detectable in cyst fluid before they reach measurable concentrations in the blood, although whether this translates into earlier clinical detection remains to be demonstrated.

These findings should nonetheless be read with care, since the strength of a signal in cyst fluid does not translate directly into clinical usefulness. That a protein, cytokine, or metabolite is more concentrated in cyst fluid than in serum reflects its local origin, but tells us little on its own about whether it can guide diagnosis. Nor does separating malignant from benign lesions within a study cohort guarantee value in practice: most of these studies contrast established cancers with clearly benign lesions, a comparison that assumes a far higher likelihood of malignancy than clinicians face when assessing an ordinary adnexal mass. In that everyday, low-prevalence setting, even a marker performing impressively in the laboratory (haptoglobin measured by SERS, for instance, at 94% sensitivity and 91% specificity) would identify far fewer true cancers among its positive results, and its ability to resolve the genuinely difficult question, borderline versus benign, has scarcely been tested. It is also worth keeping distinct the several roles a marker might play: distinguishing lesions at the time of surgery is not the same as predicting a patient’s outcome, nor as detecting disease early enough to change management, and each of these would need its own prospective study to establish. The performance figures cited throughout this review are therefore best understood as biological associations observed in selected cohorts, rather than as proven clinical tools.

The recurring suggestion that integrating proteomic, glycoproteomic, metabolomic, and genomic data offers the most promising way forward warrants a more measured treatment than it is usually given. The appeal is real, but so are the obstacles. Almost all of the cyst-fluid studies discussed here rest on small, single-centre cohorts, frequently without independent validation, as summarised in Table 2. Critically, no cyst-fluid multi-marker or multi-omics panel has yet been validated in a truly independent, external cohort. Even the most rigorously assessed candidates have been confirmed only within the originating institution. The apolipoprotein C-III and protein C inhibitor combination, and the serum amyloid A4 and astacin-like metalloendopeptidase pair, for example, were re-tested in further samples from the same centre rather than in an independent population. This distinction matters, because internal re-testing controls for assay reproducibility but not for the cohort-, centre- and workflow-specific effects that most often cause biomarkers to fail on true external validation. Combining several high-dimensional datasets in such settings invites overfitting, in which a model captures the idiosyncrasies of a particular sample set rather than generalisable biology, so that apparently strong performance can evaporate on external testing. The consequences are not merely theoretical: in one cyst-fluid study, a machine-learning classifier discriminated tumour subtypes with an area under the curve of 0.96 in cyst fluid yet only 0.57 when the same markers were measured in serum, illustrating how readily such models fail to transfer even between compartments in the same patients [39]. Histological heterogeneity compounds the difficulty, since serous, mucinous, endometrioid, and clear-cell tumours differ in their molecular profiles, and a panel optimised on one histotype need not hold for another. Layered onto these are the familiar technical confounders of multi-omics work, namely batch effects and the differences in cyst-fluid collection, processing, and storage discussed earlier, which remain largely unstandardised across studies, a limitation echoed in systematic reviews of ovarian-cancer metabolomics and of circulating tumour DNA, both of which found that cohort heterogeneity and absent external validation constrained their conclusions [36,47,48]. It bears stating plainly that increasing the number of measured features does not, in itself, produce a better clinical biomarker; reproducibility and parsimony generally matter more than breadth.

Practical considerations reinforce this caution. A test combining proteomic, glycomic, metabolomic, and genomic analysis of cyst fluid would require specialised platforms, considerable technical expertise, extended turnaround times, and substantial cost, placing it beyond routine reach in most healthcare systems [51]. A more realistic route to translation follows the established phases of biomarker development, from discovery through analytical verification and clinical validation to assessment of clinical utility [51,56]. In practice this means beginning not with a comprehensive multi-omics panel but with a small number of the most robust candidates measurable by clinically deployable assays: for cyst fluid, plausible first-step panels would pair CA125 with one or two accessible protein markers (for example haptoglobin, measurable by a rapid point-of-care assay, or apolipoprotein C-III by ELISA), or combine a targeted somatic-mutation assay with a single protein marker. Such a panel would first be locked to a pre-specified algorithm and threshold, then evaluated by multiple-reaction monitoring or immunoassay, methods that transfer between laboratories far more readily than discovery mass spectrometry, in an adequately powered, multi-centre cohort reflecting the real distribution of benign, borderline and malignant disease. Only markers surviving this step would justify expansion toward broader multi-omics integration. Analogous staged strategies, moving from omics discovery to a small panel measurable by ELISA or targeted mass spectrometry, have been applied successfully in other cancers and provide a template that cyst-fluid research has yet to follow. Consistent with this, dedicated reviews of multi-omics in ovarian cancer distinguish the small number of markers that have been validated in independent cohorts from the much larger set of candidates emerging from multi-omics discovery, and emphasise that robust quality control and large-scale, appropriately powered validation are prerequisites before clinical implementation [57]. Understood in this way, multi-omics is better regarded as a discovery strategy for identifying and prioritising candidates than as a ready-made clinical test.

A central question for clinical translation is how cyst fluid would be obtained in practice without increasing patient risk. Two routes are possible: intraoperative sampling of fluid from a cyst already being removed, and preoperative ultrasound-guided fine-needle aspiration. Intraoperative sampling carries essentially no additional risk, since the cyst is being excised regardless, but it cannot inform the decision of whether to operate, which is the very decision cyst-fluid analysis is intended to improve. Preoperative aspiration is therefore the more clinically transformative but also the more contentious route.

The principal safety concern with preoperative aspiration is iatrogenic tumour spill: puncturing an unrecognised malignant cyst could seed the peritoneum, and because preoperative (“subacute”) spill allows a longer interval for implantation than intraoperative rupture, it has been argued to carry a worse prognosis [58]. This concern is compounded by the unreliability of cyst-fluid cytology, which cannot exclude malignancy [28,58]. On this basis, aspiration of any lesion with imaging features suspicious for malignancy is inadvisable. The risk is not, however, unique to aspiration: intraoperative cyst rupture is common during laparoscopic cystectomy, so rupture is to some degree inherent to ovarian cyst management rather than exclusive to needle sampling [14]. In carefully selected, sonographically benign-appearing cysts, stratified using validated models such as the IOTA Simple Rules, ultrasound-guided aspiration has been performed with low complication and recurrence rates [14], including in defined settings such as endometrioma in pregnancy [59]. Molecular analysis of cyst fluid would therefore be most defensibly deployed as an adjunct within a low-risk, IOTA-stratified pathway, not as a licence to aspirate lesions of uncertain malignant potential. Beyond the risk of tumour spill, preoperative aspiration carries the general risks of any transvaginal needle procedure, such as bleeding, pelvic infection and injury to adjacent structures, although these are recognised but uncommon under ultrasound guidance [60]. A recent retrospective study of transvaginal sonography-guided aspiration in twenty-two women with sonographically benign ovarian cysts, selected using the IOTA Simple Rules, reported that the procedure was technically feasible in 95% of cases, was performed under local anaesthesia in all but one patient, and produced no major intra- or post-interventional complications, with a single short-term recurrence (4.5%) [14]. A concern specific to malignant lesions is dissemination of tumour cells along the needle tract or into the peritoneal cavity; the possibility of promoting peritoneal metastasis is precisely why diagnostic needle sampling of a suspected ovarian malignancy is generally avoided [58]. On feasibility, transvaginal cyst aspiration is technically simple, repeatable and well tolerated, and is already used in selected circumstances such as in women unfit for surgery, during pregnancy, or before assisted reproduction; it is not, however, part of the routine assessment of an adnexal mass, and the authors of that study concluded only that it is safe and feasible for carefully selected benign ovarian cysts. Its incorporation as a diagnostic step would therefore require prospective evaluation of both safety and added value in appropriately selected, low-risk lesions.

In such a pathway, cyst-fluid molecular analysis would complement rather than replace imaging and serum markers: a lesion judged low-risk on ultrasound and CA125 but positive for a tumour-specific mutation or biomarker signature could be triaged toward prompt surgery, whereas concordant low-risk findings might support conservative follow-up, sparing surgery in women for whom fertility preservation or surgical risk is a priority. Where imaging already indicates likely malignancy, intraoperative sampling and standard oncological surgery remain appropriate, with cyst-fluid genotyping serving to refine histotype-directed management rather than to decide whether to operate. An alternative that circumvents the aspiration dilemma entirely is to validate, in peripheral blood, biomarkers first identified in cyst fluid. Realising either pathway will require prospective studies, ideally comparing preoperative-aspiration cohorts with matched non-aspirated controls for both diagnostic yield and oncological safety, before cyst-fluid analysis can enter routine practice.

Despite these promising findings, limitations should be considered. First, many studies investigating ovarian cyst fluid involve relatively small patient cohorts, limiting the generalisation of findings [37]. Second, methodological variability in cyst fluid collection, processing and storage introduces potential pre-analytical bias, complicating comparison between studies [47]. Third, many reported biomarkers remain exploratory and have not yet undergone large-scale validation or prospective clinical evaluation. Consequently, no cyst-fluid biomarker or molecular signature has yet demonstrated sufficient clinical utility to replace or substantially outperform current diagnostic approaches based on CA125 and imaging modalities [25,61].

Through reviewing the literature pertaining to cyst fluid, certain protocols and techniques seem to provide more robust results. Wider, more integrative techniques often yielded better-validated results and uncovered more candidate signals, allowing signatures to be found and tested rather than visualising only one or a few molecules. Proteomics and metabolomics allow for broader visualisation and can be followed by validation techniques such as ELISA and immunoblotting, allowing potential biomarkers to be thoroughly tested [7,9,41]. Therefore, future research should prioritise large-scale validation studies, namely integrative multi-omics approaches combining proteomic, glycoproteomic, metabolomic and genomic data, which allow for the discovery of both proteins and metabolites that may be differentially expressed between different lesions [10,47,48].

Additionally, the development of standardised protocols for cyst fluid collection and analysis is imperative to enable reliable biomarker discovery and validation. There remains considerable potential to further investigate candidate proteins and metabolites implicated in ovarian tumourigenesis [47,48]. Building on existing findings is essential to validating new biomarkers for both diagnosis and prognosis.

In parallel, translational research is needed to define the ideal protocol for clinical use of cyst fluid. Many women undergo unnecessary surgery for lesions that ultimately prove to be non-malignant, highlighting the need for more precise and minimally invasive diagnostic tools [1,2,4]. Aspiration of cyst fluid can result in fluid leakage within the ovary, which can lead to adverse outcomes such as the proliferation of malignant cells [12,16,17]. If differential expression or signatures were to be found, the next step for researchers would be navigating the ideal way to test cyst fluid acquired in vivo prior to surgery on patients. Alternatively, if a potential diagnostic biomarker is identified within cyst fluid, its expression within serum levels could be deemed significant due to the known activity in the cyst fluid. This methodology could circumvent the potential difficulties with cyst fluid aspiration and continue using serum for patient diagnosis.

## 6. Conclusions

Lying in direct contact with the lesion, ovarian cyst fluid concentrates tumour-derived molecules and may offer a window onto tumour-microenvironment interactions relevant to progression and metastasis. Proteomic, glycoproteomic, inflammatory, metabolic, and genomic studies collectively demonstrate its capacity to capture early and mechanistically relevant alterations across benign, borderline, and malignant lesions. Although individual markers remain insufficient for standalone clinical implementation, combining complementary molecular layers may offer a more promising approach, although this remains to be demonstrated in prospective studies. Future efforts must prioritise standardisation, validation, and translational feasibility to realise the diagnostic and biological potential of ovarian cyst-fluid analysis.

## Figures and Tables

**Table 1:** Ovarian tumour classification according to the World Health Organization (WHO) 2020 classification [24].

Epithelial	Mesenchymal	Sex Cord Stromal	Germ Cell
Serous Mucinous Endometrioid Clear cell tumours Seromucinous Brenner tumours Undifferentiated and dedifferentiated carcinoma Mesonephric-like adenocarcinoma Carcinosarcoma Mixed carcinoma	Endometrial stromal sarcoma Smooth muscle tumours Ovarian myxoma	Pure stromal tumours Pure sex cord tumours Mixed sex cord stromal tumours	Benign teratoma Immature teratoma, NOS Dysgerminoma Yolk sac tumour Embryonal carcinoma Choriocarcinoma NOS Mixed germ cell tumour Monodermal teratomas and somatic-type tumours arising from a dermoid cyst Mixed germ cell sex cord stromal tumours

**Table 2:** Summary of studies analysing molecular and biochemical markers in ovarian cyst fluid, grouped by molecular class. Diagnostic-performance values are given only where the original study reported them.

Study [Ref]	Biomarker(s)	Class	Cohort and Comparison	Method	Main Finding (Performance)	Validation/Limitations
Proteomic and glycoproteomic						
Kristjansdottir 2012 [7]	ApoC-III, PCI (+serum CA125)	Proteomic	Cyst fluid + serum; benign vs. malignant EOC (*n* = 192: 129/63); mixed epithelial	SELDI-TOF MS; ELISA	ApoC-III and PCI independent predictors; with CA125, AUC 0.94, specificity 68%→88%	Validated (ELISA); peaks unsequenced, PCI unconfirmed, needed CA125
Kristjansdottir 2013 [9]	SAA4	Proteomic	Cyst fluid vs. plasma; benign vs. malignant serous, stage I–II (*n* = 15 discovery; 68 + 68 validation)	iTRAQ MS; immunoblot	SAA4 higher in malignant cyst fluid (*p* = 0.001), not plasma; 87/837 proteins differential	Validated (immunoblot); serous-only discovery; negative in most mucinous
Skaggs 2013 [37]	Calgranulin A, calgranulin B	Proteomic	Cyst fluid; benign vs. borderline vs. malignant; mixed epithelial	SELDI-TOF MS	Higher in borderline/malignant; early-stage sensitivity 28.6–63.6% at 95% specificity; accuracy 74.8%	Exploratory (no external validation); not ovarian-specific
Poersch 2016 [38]	APOE (+SERPINF2, SERPING1, ADAM17, CD44, OVGP1)	Proteomic	Tumour fluid; benign serous cystadenoma vs. high-grade serous carcinoma (10 vs. 10 pooled)	LC-MS; MRM; Western blot	75/1135 proteins differential; 6 significant; APOE confirmed higher in malignant	Validated (MRM, Western blot); small pooled discovery; no sensitivity/specificity
Marcišauskas 2019 [39]	Panel (TALDO1, ALDOA, TKT1, CP, MSLN, CLU, TNXB, LAMC1, MUC16)	Proteomic (ML panel)	Cyst fluid + serum; benign serous vs. type I vs. type II (all stage I; *n* = 27: 9/9/9)	TMT-LC-MS/MS; machine learning	AUC 0.96 in cyst fluid vs. 0.57 in serum (only ALDOA significant)	Verified (both fluids, same study); very small, stage-I-only; no external cohort
Perumal 2019 [31]	Haptoglobin	Proteomic	Cyst fluid; benign vs. malignant EOC (*n* = 111: 57/54); predominantly serous	SERS assay (<10 min)	Sensitivity 94%, specificity 91%, AUC 0.966; outperformed CA125 (85%/90%)	Validated vs. histology (single centre); retrospective; stage-4 subgroup n = 7
Ahmad 2015 [40]	35 kDa protein (and 100 kDa)	Proteomic	Serum + cyst fluid; malignant vs. benign vs. controls (*n* = 50: 10/10/30)	SDS-PAGE; ELISA	35 kDa band unique to malignant serum/cyst fluid; 100 kDa absent in malignancy	Exploratory (gel-based); proteins unsequenced; dated method
**Metabolomic**						
Kyriakides 2016 [41]	Lactate, choline, citrate, lysine, hypoxanthine	Metabolomic	Cyst fluid; benign vs. borderline vs. malignant epithelial (*n* = 23); mixed epithelial	^1^H NMR spectroscopy	Citrate higher in benign, lysine in malignant (*p* < 0.05); choline/lactate rose benign→malignant; hypoxanthine in a malignant subset	Exploratory (single cohort, no validation)
**Inflammatory and angiogenic**						
Daraï 2003 [8]	IL-6, IL-8, TNF-α	Inflammatory	Serum + cyst fluid; endometrioma vs. benign vs. malignant (*n* = 77: 34/30/13)	Immunoradiometric assay	All higher in cyst fluid than serum, and higher in malignant	Exploratory; small malignant group
Pawlik 2021 [42]	IL-6, IL-8, TNF-α	Inflammatory	Serum; ovarian carcinoma vs. benign cystic lesions	ELISA; ROC analysis	IL-8 and TNF-α, alone and combined, discriminated malignant from benign	Exploratory (not externally validated)
Fasciani 2001 [43]; Artini 2008 [44]	VEGF, sVEGFR-1	Angiogenic	Cyst fluid, serum, peritoneal; cystadenoma vs. endometrioma vs. carcinoma (*n* = 48: 14/28/6)	ELISA; Western blot	VEGF higher in endometrioma/carcinoma vs. cystadenoma; VEGF:sVEGFR-1 ratio highest in cancer; serum unchanged (local)	Exploratory (mechanistic); small malignant group (*n* = 6), advanced-stage
van Tilborg 2014 [45]	Angiostatin, plasminogen (uPA, tPA)	Angiogenic	Cyst fluid; benign, malignant, functional, dermoid, endometriotic	Western blot; ELISA	Plasminogen→angiostatin conversion via plasminogen activators; angiostatin across cyst types, no tumour-type correlation	Exploratory (mechanistic); semiquantitative
**Glycoproteomic**						
Kuzmanov 2013 [10]	Sialylated glycoproteins (21 candidates: AMBP, CDH6, POSTN, KLK6, etc.)	Glycoproteomic	Malignant cyst fluid/ascites vs. benign cyst fluid/effusion vs. cell lines (pooled: 14/13/10/20/4)	Lectin + hydrazide enrichment; ESI-LTQ-Orbitrap MS	333 proteins, 579 sialylation sites; 21 candidates unique to cancer-associated fluids	Exploratory (candidate discovery); pooled, advanced-stage
Ruma 2025 [30]	CA19-9 glycovariants (MUC1, MUC16, sialyl-Tn)	Glycoproteomic	Cyst fluid + serum; benign vs. borderline vs. malignant epithelial	Nanoparticle glycovariant assay	Glycan-context CA19-9 discriminated borderline/malignant better than conventional CA19-9 or CA125	Validated within study; single study; needs external validation
**Genomic**						
Wang 2016 [12]	Tumour-derived DNA (TP53, KRAS, BRAF, others)	Genomic	Cyst fluid; benign/non-neoplastic vs. borderline vs. type I/II cancer (*n* = 77: 10/12/24/31)	Safe-SeqS (17-gene panel)	Mutations in 83% borderline, 77% type I, 100% type II; absent in benign; sensitivity 87%, specificity 100% (surgery-requiring)	Validated (tumour-matched, independent PCR); small benign group; misses some type I/borderline
**Conventional biomarkers and cytology**						
Kolwijck 2010 [11]	CA125	Conventional antigen	Cyst fluid + serum; EOC (*n* = 54); mixed epithelial	CA125 immunoassay	Cyst-fluid CA125 ~300× serum (median 55,500 vs. 179 U/mL, *p* < 0.001); weak correlation (R = 0.337); no independent prognostic value	Retrospective, single-centre
Chudecka-Głaz 2008 [34]	FSH (and LH)	Hormonal	Serum vs. tumour/cyst fluid vs. pouch fluid; cancer/borderline/benign/non-neoplastic (*n* = 277)	Microparticle enzyme immunoassay	Fluid FSH ≈ serum in malignant/borderline (no barrier); far lower than serum in benign (6.93 vs. 28.96) and non-neoplastic (*p* < 10^−6^)	Consistent with prior reports; mechanistic, no diagnostic metrics
Podzielinski 2013 [35]	Apolipoproteins (apoA2, apoE)	Proteomic	Cyst fluid; malignant vs. benign/borderline; mixed epithelial	SELDI-TOF MS; ELISA	Elevated in malignant; apoA2/apoE classified malignant at sensitivity 55.1%, specificity 95%, accuracy 88.1%	Validated (ELISA); modest sensitivity
Zhou 2018 [28]	Fine-needle aspiration cytology	Cytology	Cyst fluid; benign vs. malignant (*n* = 459 FNA specimens, 18 years)	FNA cytology	Sensitivity 54%, specificity 100%, PPV 1.00, NPV 0.97 (prevalence 7.3%); missed 33% malignant, 69% borderline	Large retrospective validation vs. histology
Parker 1999 [46]	Lactate dehydrogenase, glucose, protein	Biochemical	Cyst fluid; non-neoplastic vs. benign vs. malignant (*n* = 58: 15/23/20)	Enzymatic/biochemical assays	LDH ≥ 451 U/L: sensitivity 90%, specificity 71%, PPV 64%; measurable in only 1/3 of endometriomas (debris)	Exploratory; non-specific; unmeasurable in viscous/endometriotic samples

Validation status distinguishes candidates confirmed by an independent method or cohort (“validated”), those confirmed only within the original study (“verified”), and single-cohort discovery or mechanistic findings (“exploratory” or “mechanistic”). Abbreviations: ADAM17, a disintegrin and metalloproteinase 17; ALDOA, aldolase A; AMBP, alpha-1-microglobulin/bikunin precursor; ApoC-III, apolipoprotein C-III; apoA2/apoE, apolipoprotein A-II/E; APOE, apolipoprotein E; AUC, area under the curve; BRAF, B-Raf proto-oncogene; CA19-9/CA125, cancer antigen 19-9/125; CD44, cluster of differentiation 44; CDH6, cadherin-6; CLU, clusterin; CP, ceruloplasmin; EOC, epithelial ovarian cancer; ELISA, enzyme-linked immunosorbent assay; ESI-LTQ, electrospray ionisation linear trap quadrupole; FNA, fine-needle aspiration; FSH, follicle-stimulating hormone; IL, interleukin; iTRAQ, isobaric tags for relative and absolute quantitation; KLK6, kallikrein-6; KRAS, Kirsten rat sarcoma viral oncogene homolog; LAMC1, laminin subunit gamma-1; LC-MS, liquid chromatography–mass spectrometry; LDH, lactate dehydrogenase; LH, luteinising hormone; ML, machine learning; MRM, multiple-reaction monitoring; MS, mass spectrometry; MSLN, mesothelin; MUC1/MUC16, mucin 1/16; NMR, nuclear magnetic resonance; NPV, negative predictive value; OVGP1, oviductal glycoprotein 1; PCI, protein C inhibitor; POSTN, periostin; PPV, positive predictive value; ROC, receiver-operating characteristic; SAA4, serum amyloid A4; SDS-PAGE, sodium dodecyl-sulfate polyacrylamide gel electrophoresis; SELDI-TOF, surface-enhanced laser desorption/ionisation time-of-flight; SERPINF2/G1, serpin family F member 2/G member 1; SERS, surface-enhanced Raman spectroscopy; sVEGFR-1, soluble vascular endothelial growth factor receptor-1; TALDO1, transaldolase 1; TKT1, transketolase 1; TMT, tandem mass tag; TNF-α, tumour necrosis factor-alpha; TNXB, tenascin-XB; TP53, tumour protein p53; tPA/uPA, tissue/urokinase plasminogen activator; VEGF, vascular endothelial growth factor.

## Data Availability

No new data were created or analysed in this study. Data sharing is not applicable to this article.

## Abbreviations

The following abbreviations are used in this manuscript:

ADAM17	a disintegrin and metalloproteinase 17
ALDOA	aldolase A
AMBP	alpha-1-microglobulin/bikunin precursor
ApoA2	apolipoprotein A-II
ApoC-III	apolipoprotein C-III
APOE	apolipoprotein E
AUC	area under the receiver-operating-characteristic curve
BRAF	B-Raf proto-oncogene
CA125	cancer antigen 125
CA19-9	cancer antigen 19-9
CCL2	C-C motif chemokine ligand 2
CD44	cluster of differentiation 44
CDH6	cadherin-6
cGAS	cyclic GMP-AMP synthase
CLU	clusterin
CP	ceruloplasmin
CT	computed tomography
ctDNA	circulating tumour DNA
DNA	deoxyribonucleic acid
EAOC	endometriosis-associated ovarian carcinoma
ELISA	enzyme-linked immunosorbent assay
EOC	epithelial ovarian cancer
ESI-LTQ	electrospray ionisation linear trap quadrupole
FIGO	International Federation of Gynecology and Obstetrics
FNA	fine-needle aspiration
FSH	follicle-stimulating hormone
GRO-α	growth-regulated oncogene-alpha
Hb	haemoglobin
hCG	human chorionic gonadotropin
HE4	human epididymis protein 4
IHC	immunohistochemistry
IL-6	interleukin-6
IL-8	interleukin-8
IOTA	International Ovarian Tumour Analysis
IRS1	insulin receptor substrate 1
ITIH4	inter-alpha-trypsin inhibitor heavy chain H4
iTRAQ	isobaric tags for relative and absolute quantitation
IVF	in vitro fertilisation
KLK6	kallikrein-6
KRAS	Kirsten rat sarcoma viral oncogene homolog
LAMC1	laminin subunit gamma-1
LC-MS	liquid chromatography–mass spectrometry
LDH	lactate dehydrogenase
LH	luteinising hormone
MCP-1	monocyte chemoattractant protein-1
MEK	mitogen-activated protein kinase kinase (MAPK/ERK kinase)
metHb	methaemoglobin
miR-145	microRNA-145
ML	machine learning
MMP	matrix metalloproteinase
MR	magnetic resonance
MRI	magnetic resonance imaging
MRM	multiple-reaction monitoring
MS	mass spectrometry
MSLN	mesothelin
MUC1	mucin 1
MUC16	mucin 16
NMR	nuclear magnetic resonance
NOS	not otherwise specified
NPV	negative predictive value
NRAS	neuroblastoma RAS viral oncogene homolog
OVGP1	oviductal glycoprotein 1
oxyHb	oxyhaemoglobin
PCI	protein C inhibitor
PCOS	polycystic ovary syndrome
PCR	polymerase chain reaction
PI3K	phosphoinositide 3-kinase
PIK3R1	phosphoinositide-3-kinase regulatory subunit 1
POSTN	periostin
PPP2R1A	protein phosphatase 2 scaffold subunit A-alpha
PPV	positive predictive value
PTEN	phosphatase and tensin homolog
RAF	rapidly accelerated fibrosarcoma kinase
RAS	rat sarcoma viral oncogene homolog
RMI	Risk of Malignancy Index
ROC	receiver-operating characteristic
ROMA	Risk of Ovarian Malignancy Algorithm
ROS	reactive oxygen species
SAA4	serum amyloid A4
Safe-SeqS	Safe-Sequencing System
SDS-PAGE	sodium dodecyl-sulfate polyacrylamide gel electrophoresis
SELDI-TOF	surface-enhanced laser desorption/ionisation time-of-flight
SERPINA1	serpin family A member 1
SERPINF2	serpin family F member 2
SERPING1	serpin family G member 1
SERS	surface-enhanced Raman spectroscopy
STING	stimulator of interferon genes
STn	sialyl-Tn antigen
sVEGFR-1	soluble vascular endothelial growth factor receptor-1
TALDO1	transaldolase 1
TCA	tricarboxylic acid
TIMP	tissue inhibitor of metalloproteinases
TKT1	transketolase 1
TME	tumour microenvironment
TMT	tandem mass tag
TMT-LC-MS/MS	tandem mass tag liquid chromatography–tandem mass spectrometry
TNF-α	tumour necrosis factor-alpha
TNXB	tenascin-XB
TP53	tumour protein p53
tPA	tissue plasminogen activator
uPA	urokinase plasminogen activator
VEGF	vascular endothelial growth factor
WHO	World Health Organization
